# Blood Composite Scores in Patients with Systemic Lupus Erythematosus

**DOI:** 10.3390/biomedicines11102782

**Published:** 2023-10-13

**Authors:** Júlia Mercader-Salvans, María García-González, Juan C. Quevedo-Abeledo, Adrián Quevedo-Rodríguez, Alejandro Romo-Cordero, Soledad Ojeda-Bruno, Fuensanta Gómez-Bernal, Raquel López-Mejías, Candelaria Martín-González, Miguel Á. González-Gay, Iván Ferraz-Amaro

**Affiliations:** 1 Division of Dermatology, Hospital Universitario de Canarias, 38320 Tenerife, Spain; juliamercader96@gmail.com; 2 Division of Rheumatology, Hospital Universitario de Canarias, 38320 Tenerife, Spain; margagon23@hotmail.com; 3 Division of Rheumatology, Hospital Doctor Negrín, 35010 Las Palmas de Gran Canaria, Spain; quevedojcarlos@yahoo.es (J.C.Q.-A.); adrian-ce@hotmail.es (A.Q.-R.); soas@comlp.es (S.O.-B.); 4 Division of Internal Medicine, Hospital Universitario de Canarias, 38320 Tenerife, Spain; alexromo96co@gmail.com (A.R.-C.); mmartgon@ull.edu.es (C.M.-G.); 5 Division of Central Laboratory, Hospital Universitario de Canarias, 38320 Tenerife, Spain; fuensanta95@gmail.com; 6 Epidemiology, Genetics and Atherosclerosis Research Group on Systemic Inflammatory Diseases, Instituto de Investigación Sanitaria Marqués de Valdecilla (IDIVAL), 39011 Santander, Spain; rlopezmejias78@gmail.com; 7 Department of Internal Medicine, Universidad de La Laguna (ULL), 38200 Tenerife, Spain; 8 Division of Rheumatology, IIS-Fundación Jiménez Díaz, 28040 Madrid, Spain; 9 Department of Medicine, University of Cantabria, 39005 Santander, Spain; 10 Cardiovascular Pathophysiology and Genomics Research Unit, School of Physiology, Faculty of Health Sciences, University of the Witwatersrand, Johannesburg 2000, South Africa

**Keywords:** hematological composite scores, systemic inflammation response index, systemic lupus erythematosus

## Abstract

Complete blood count-derived ratios have been described as inflammatory biomarkers in several diseases. These hematological scores include the neutrophil-to-lymphocyte ratio (NLR), monocyte-to-lymphocyte ratio (MLR), platelet-to-lymphocyte ratio (PLR), and systemic immune-inflammatory index ([SIRI]; neutrophils × monocytes/lymphocytes). Our aim was to study how these biomarkers are related to disease expression in a large and well-characterized series of patients with systemic lupus erythematosus (SLE). A total of 284 SLE patients and 181 age- and sex-matched healthy controls were recruited. The NLR, MLR, PLR, and SIRI were calculated, and activity (SLEDAI-2K), severity (Katz), and damage index (SLICC-DI) scores were assessed in patients with SLE. Multivariable linear regression analysis was performed to study whether these scores differ between patients and controls and how they are related to clinical and laboratory features of the disease. Crude cell counts of neutrophils, monocytes, lymphocytes, and platelets were lower in SLE patients compared to controls. Despite this, NLR, MLR, and PRL, but not SIRI, were higher in SLE patients than in controls after multivariable analysis. However, the relationship between the different scores and disease characteristics was limited. Only the Katz severity index revealed a significant positive relationship with SIRI, NLR, and MLR after adjustment for covariates. Similarly, alternative complement cascade activation and low C3 were significantly associated with higher NLR, MLR, and PLR. In conclusion, although cytopenias are a common feature of patients with SLE, hematologic composite scores are independently higher in this population compared to controls. However, the relationship of these scores with the characteristics of the disease is scarce, with the relationship with the complement system being the most consistent.

## 1. Introduction

Complete blood count results are routinely used to assess acute or chronic infections and inflammation. This is because white blood cells and platelets are found within the chronic inflammatory environment, playing a role in the release of cytokines, proteases, angiogenic factors, and chemokines [1]. Consequently, several blood composite scores have been proposed as biomarkers of systemic inflammation. These include the neutrophil–lymphocyte ratio (NLR) [2], the platelet–lymphocyte ratio (PLR) [3], and the lymphocyte–monocyte ratio (LMR) [4]. Additionally, a new inflammation-related biomarker called the systemic inflammation response index (SIRI) was released in 2016 and is computed as neutrophils × monocytes/lymphocytes [5]. All of them have been proposed as highly sensitive markers of inflammation in various inflammatory disorders and other conditions, such as cancer, cardiovascular disease, or infection [6,7,8].

Systemic lupus erythematosus (SLE) is a chronic autoimmune disease of unknown cause that can affect virtually any organ of the body. The etiology of SLE remains unknown and is clearly multifactorial. Many observations suggest a role for genetic [9], hormonal [10], immunologic [11], and environmental factors [12]. It is clear that several of the clinical manifestations are mediated directly or indirectly by antibody formation and the creation of immune complexes [13]. Accordingly, immunologic abnormalities, especially the production of several antinuclear antibodies, are a prominent feature of the disease. However, other pathogenic mechanisms are involved, such as gene mutations, complement deficiencies that lead to the defective clearance of dying cells (a mechanism also called efferocytosis), mutations in extracellular DNAses, and defective interferon signaling [14]. Furthermore, SLE is characterized by many clinical symptoms and different pathophysiological abnormalities. In this sense, hematological abnormalities are frequent in SLE, both at the time of diagnosis and throughout the course of the disease. The main hematologic manifestations of SLE include anemia, leukopenia, thrombocytopenia, lymphadenopathy, splenomegaly, and/or macrophage activation syndrome [15]. Remarkably, elevation in acute phase reactants is not a typical characteristic of patients with SLE [16], and when they increase, their increment is usually modest [17]. Additionally, other markers of inflammation, such as white blood cell and platelet counts, albumin, and complement levels, are frequently altered by the disease itself, making them unreliable indicators of an acute phase response in SLE. Since blood composite scores have been related to systemic inflammation, their study in SLE is pertinent because they could become markers of disease activity. Some evidence exists regarding their role in SLE. For example, basophil count has been described to be lower in SLE children compared to controls [18]. Similarly, the absolute counts and frequencies of natural killer T-like cells were described to be downregulated in SLE patients significantly, which correlated to disease activities and could recover to normal after treatment [19].

The behavior of the composite blood scores has not been fully studied in patients with SLE. For this reason, in the present work, we have evaluated these hematological scores in a large series of patients and controls. The aim of our work was to compare the values between both populations and to analyze the relationship of these composite hematology panel scores to a wide range of disease manifestations, including disease activity, damage, severity scores, and complement system values.

## 2. Materials and Methods

### 2.1. Study Participants

All SLE patients were 18 years of age or older, possessed a clinical diagnosis of SLE, and fulfilled at least 4 classification criteria for SLE as defined by the American College of Rheumatology (ACR) [20]. These patients had been diagnosed by rheumatologists and were consistently monitored in rheumatology outpatient clinics. Patients were recruited from January 2016 to November 2021. Inclusion criteria permitted participation for SLE patients taking prednisone, as long as the equivalent dose was ≤10 mg/day, considering the common use of glucocorticoids in SLE treatment. Controls were selected from the community and were recruited by general practitioners at primary care centers. However, individuals with a history of any inflammatory rheumatic disease were excluded from the control group. Notably, none of the control participants were undergoing glucocorticoid therapy. This research adhered to the principles outlined in the Declaration of Helsinki. The study protocol gained approval from the Institutional Review Committee at Hospital Universitario de Canarias and at Hospital Universitario Doctor Negrín, both located in Spain. Additionally, all subjects provided their informed written consent to participate in the study (Approval Number 2015_84).

### 2.2. Data Collection

Participants in the study completed a survey regarding cardiovascular risk factors and their use of medications. They also underwent a comprehensive physical examination conducted under standardized conditions. Measurements were taken for weight, height, body mass index, waist circumference, as well as systolic and diastolic blood pressure (measured while participants were lying down). Smoking habits and hypertension treatment information were collected from the survey. Detailed medical records were carefully reviewed to confirm specific diagnoses and prescribed medications. The activity and damage caused by the disease were assessed using the Systemic Lupus Erythematosus Disease Activity Index-2000 (SLEDAI-2K) [21] and the Systemic Lupus International Collaborating Clinics/American College of Rheumatology (SLICC/ACR Damage Index -SDI-) [22], respectively. For this study, the SLEDAI-2K index was divided into none (0 points), mild (1–5 points), moderate (6–10 points), high (11–19), and very high activity (>20) as previously defined [23]. The severity of the disease was measured using the Katz index [24].

Moreover, participants with SLE underwent carotid ultrasonography to measure the thickness of the carotid artery’s intima-media wall (cIMT) in the common carotid artery. The presence of focal plaques in the extracranial carotid artery was also identified, following the Mannheim consensus definitions [25]. Dyslipidemia was defined as meeting one or more of the following criteria: total cholesterol > 200 mg/dL, triglycerides > 150 mg/dL, HDL-cholesterol < 40 mg/dL in men or <50 mg/dL in women, or LDL-cholesterol > 130 mg/dL. Hematological composite scores were calculated as follows: neutrophil-to-lymphocyte ratio (NLR) = neutrophils/lymphocytes; monocyte-to-lymphocyte ratio (MLR) = monocytes/lymphocytes; platelet-to-lymphocyte ratio (PLR) = platelets/lymphocytes; systemic inflammation response index (SIRI) = neutrophils × monocytes/lymphocytes. Neutrophils, monocytes, lymphocytes, and platelets were measured per 1000 cells/μL, except for platelets, which were measured per 100,000 cells/μL. Blood samples were collected after fasting.

### 2.3. Complement System Assessment

SVAR Functional Complement Assays offered under the Wieslab^®^ brand in Sweden (SVAR Life Science, Malmö, Sweden) are utilized to evaluate the activity of the classical, alternative, and lectin pathways within the complement system. These assays employ a combination of the hemolytic assay principle for assessing complement function along with the utilization of labeled antibodies specific to the neoantigen generated as a result of complement activation. The quantity of neoantigen produced is proportional to the functional activity of the complement pathways. In these assays, microtiter strip wells are coated with activators specific to the classical, alternative, or lectin pathways. The patient’s serum is diluted using a diluent that contains a particular blocker, ensuring that only the targeted pathway is activated. During the incubation of the diluted patient serum in the wells, the specific coating triggers complement activation. Subsequently, the wells are washed, and the presence of C5b-9 is detected using an alkaline phosphatase-labeled specific antibody directed against the neoantigen expressed during the formation of the membrane attack complex (MAC). Following an additional washing step, detection of the specific antibodies is achieved through incubation with an alkaline phosphatase substrate solution. The degree of complement activation is correlated with the intensity of color and quantified in terms of absorbance (optical density). The extent of MAC formation (neo-epitope) reflects the activity of the complement cascade. The test results are semiquantitatively expressed via calculating the optical density ratio between a positive control and the sample. Notably, lower levels of classical, alternative, and lectin cascade values indicate a greater activation of the respective pathway. Wieslab^®^ has validated these functional assays through assessing their correlation and agreement with the classical CH50 and AH50 hemolytic tests (https://www.svarlifescience.com/ accessed on 1 September 2023).

### 2.4. Statistical Analysis

Demographic and clinical characteristics of individuals with SLE were presented as mean values with their corresponding standard deviations or as percentages for categorical variables. For continuous variables that did not exhibit a normal distribution, data were conveyed as medians along with the interquartile range (IQR). Univariable distinctions between patients and the control group were evaluated using various statistical tests such as Student’s *t*-test, the Mann–Whitney U-test, Chi-squared test, or Fisher’s exact test, chosen based on the normality of distribution or the sample size. Disparities between SLE patients and the control group in terms of hematological scores were analyzed through multivariable linear regression analysis, employing the control group as the reference category. The relationship between disease-related information and composite blood scores (a continuous dependent variable) was investigated using multivariable linear regression analysis, with adjustments made for potential confounding variables. Confounding variables were selected from demographic factors and traditional cardiovascular risk factors if their *p*-values were below 0.20 in the univariable analysis of hematological scores. All statistical analyses were carried out utilizing Stata software, version 17/SE (StataCorp, College Station, TX, USA), and a significance level of 5% was adopted for two-sided tests. A *p*-value of less than 0.05 was regarded as indicative of statistical significance.

## 3. Results

### 3.1. Demographic Data of Patients and Controls and Data Related to the Disease of Patients with Systemic Lupus Erythematosus

Table 1 offers an overview of the characteristics of the 284 patients and 181 age- and gender-matched controls who were enrolled in the study. The majority of participants were women (over 90% in both groups), and their average age ± standard deviation was 50 ± 12 years in both cohorts. The mean body mass index was slightly lower in SLE patients compared to controls, a difference that was statistically significant (30 ± 3 vs. 28 ± 6 kg/m^2^ *p* ≤ 0.001). Traditional cardiovascular risk factors were prevalent among both patient and control groups. While diabetes prevalence was notably higher among controls, hypertension was more common among individuals with SLE. Additionally, the utilization of statins showed no significant distinction between the groups, but there was a higher intake of aspirin among SLE patients. The findings from carotid ultrasonography in SLE patients indicated a mean cIMT of 628 ± 109 microns, with 36% of patients exhibiting carotid plaques (see Table 1).

The median duration of disease for SLE patients was 16 years (with an interquartile range of 7–24 years). A significant proportion of SLE patients had either no activity (40%) or mild to moderate activity (39%), as assessed using the SLEDAI-2K score. The SLICC-SDI and Katz indices were 1 (with an interquartile range of 0–2) and 2 (with an interquartile range of 1–4), respectively. Notably, 68% of patients exhibited a SLICC-SDI score of 1 or higher. About half of the patients (50%) were using prednisone, with a median daily dosage of 5 mg/day (with an interquartile range of 5–7.5 mg/day). At the time of enrollment, 67% of patients tested positive for anti-DNA antibodies, while 69% tested positive for anti-ENA antibodies, with anti-SSA antibodies being the most frequently detected (35%). Hydroxychloroquine was being utilized by 69% of patients during the study period. Other disease-modifying antirheumatic drugs, such as methotrexate (11%) and azathioprine (15%), were less commonly employed. Further information concerning SLE is available in Table 1.

### 3.2. Multivariable Analysis of the Differences between Patients and Controls in Hematological Composite Scores

The complete count of neutrophils, lymphocytes, monocytes, and platelets was lower in patients with SLE compared to controls (Table 2). This difference was maintained after adjustment for covariates. However, despite these lower levels in crude blood count, hematological composite scores NLR (beta coef. 0.5 [95%CI 0.08–0.9], *p* = 0.020), MLR (beta coef. 0.1 [95%CI 0.02–0.2], *p* = 0.012), and PLR (beta coef. 45 [95%CI 18–81], *p* = 0.012), but not SIRI (beta coef. 0.2 [95%CI −0.1–0.5], *p* = 0.26), were significantly higher in SLE compared to controls. This finding was also observed after multivariate analysis adjusted for confounding factors (Table 2).

### 3.3. Relationship of Demographic and Disease Characteristics to Composite Blood Scores

The relationship of the different hematological scores with the characteristics of the disease is shown in Table 3. NLR and MLR revealed no relationship with demographic characteristics and cardiovascular comorbidity. SIRI was significantly lower in women and higher in obese subjects. On the other hand, PLR showed a significant negative association with age, smoking, statin use and presence of carotid plaque (Table 3).

Regarding the characteristics of SLE, no relationship was found between the hematological scores and the duration of the disease and the serum CRP levels. Similarly, SLICC and SLEDAI were not related to hematology score levels. However, the Katz index showed a positive and consistent relationship with SIRI (beta coef. 0.09 [95%CI 0.02–0.2], *p* = 0.011), NLR and MLR, but not with PLR. Anti-beta2 glycoprotein IgG was the only antibody significantly associated with SIRI. Anticardiolipin IgG and anti beta2 glycoprotein IgG antibodies were related to higher levels of NLR. On the other hand, the presence of anti-ENA and anti-RNP showed a positive relationship with higher levels of MLR and PLR. Besides, SSA and anti-nucleosome were only associated with higher levels of PLR. Finally, the presence of antiphospholipid syndrome was not associated with any of the scores.

With respect to the therapies used in the disease, the intake of azathioprine showed the highest consistent relationship with the hematological scores, being associated with higher levels of NLR, MLR, and PLR (Table 3 and Table 4).

### 3.4. Analysis of the Relationship of Complement System Pathways and Components to Blood Scores in SLE Patients

The complement pathways and the relationships of individual elements to hematology scores are illustrated in Table 5. The alternative complement pathway functional assay had a negative and significant relationship with NLR (beta coef. −0.007 [95%CI −0.01–(−0.002)], *p* = 0.007), MLR (beta coef. −0.001 [95%CI −0.002–(−0.0007)], *p* ≤ 0.001), and PLR (beta coef. −0.08 [95%CI −1–(−0.04)], *p* ≤ 0.001). That is, the greater the activation of this pathway (lower levels), the higher these scores will be. Similarly, lower levels of C3, a common element of all three pathways, were associated with higher levels of MLR (beta coef. −0.0007 [95%CI −0.001–(−0.00002)], *p* = 0.043) and PLR (beta coef. −0.04 [95%CI −0.07–(−0.06)], *p* = 0.020) (Table 5).

## 4. Discussion

Although cytopenias are a common feature of patients with SLE, we have found in our work that hematologic composite scores NLR, MLR, and PLR, but not SIRI, are independently higher in SLE patients compared to controls. Nevertheless, the relationship of these scores with characteristics of the disease, like disease activity or damage scores and acute phase reactants, is scarce. Only the association with the alternative cascade of the complement system was consistent. We believe these scores may not serve as biomarkers for disease activity or for the detection of specific organ comorbidity in SLE.

Several previous reports have studied NLR in SLE. In this sense, NLR has been shown to be higher in SLE group compared to sex-age matched control groups and to be correlated to disease activity [26,27,28,29]. NLR was also positively correlated with the SLEDAI-2K score, ESR, and CRP, whereas it was not correlated with C3 or C4 [29]. NLR has been observed in the severely active group compared to the mildly active group; to significantly positively relate to SLEDAI score, renal SLEDAI score, 24 h urine protein, cellular crescents, and tubular atrophy; and to negatively correlate with serum albumin [28]. Several other reports have associated NLR to renal disease in SLE [30,31]. Additionally, NLR has been shown to significantly decrease after treatment [28]. Additionally, NLR has been associated with severe depression and poor/fair self-rated health in SLE [32] and to be a better marker than SIRI in predicting nephritis [33]. Another report has demonstrated that NLR had correlations with serological indicators and may predict organ involvement in juvenile SLE, particularly cutaneous, arthritis, serositis, and hematological involvement [34]. An NLR cut off value of 2.94 has been proposed to determine active disease of SLE [35]. Moreover, NLR has been shown to associate with key immunopathological events, including type I interferon activity and neutrophil activation [36].

Similarly, PLR has been linked to disease activity in SLE [27,28,37]. No significant association between the course of the disease in pregnancy and PLR was documented in one report [38]. PLR has been described to be increased significantly in the lupus nephritis group without infection as compared with those in healthy controls [39]. Additionally, PLR was related only to anti ds-DNA but not to serum urea, serum creatinine, and 24 h urinary protein [40]. The best PLR cut-off value for SLE prediction has been set to 203 [41].

The role of MLR and SIRI in SLE has been less frequently studied. Similarly to other reports, MLR was described to correlate to disease activity, ESR, and CRP [42] and to be increased in SLE compared to controls [39] but to not differentiate between SLE patients with and without infection [43]. Concerning SIRI, in a retrospective study, 76 patients with SLE exhibited higher levels of SIRI, NLR, and PLR than 76 age- and gender-matched healthy controls [33]. SIRI was positively correlated with CRP but not with SLEDAI-2K.

Taken together, all these studies show that NLR and, in most cases, PLR are increased in SLE patients when compared with controls. These findings were confirmed in our study, in which a larger sample size allowed us to perform multivariable analysis considering confounding factors. However, we could not find any relationship with disease activity. This could be due to the fact that the patients in our series were mostly in low or moderate activity.

Contrary to what was previously reported, we did not observe a relationship between hematological scores and the presence of kidney disease. However, in our study, we included scores of damage or severity, as well as a detailed description of the items in each score. In this sense, we observed a relationship between SIRI, NLR, and MLR, but not PLR, and the Katz severity index. This relationship held after adjusting for covariates. However, a detailed analysis of the items on this score revealed that the relationship of MLR and LPR was primarily with the low-hematocrit item. Given that the presence of anemia has a high weight in this index, we believe that the relationship of blood scores with the Katz index should be interpreted with caution.

The activation of the complement system has been associated with cytopenias in SLE. For example, the presence of complement on the surface of red blood cells is generally associated with some degree of anemia [44], whereas neutropenia is thought to be induced by immune abnormalities, including immune complexes leading to complement activation [45]. A close interplay between the complement system and platelets has also been described [46]. In addition, lymphopenia, especially involving regulatory T cells, has been observed in 20 to 75 percent of SLE patients, particularly during active disease [44]. As a novelty, in our study we carried out a complete characterization of the complement system that included functional tests as well as the evaluation of individual elements of the three pathways. We found that low levels of the alternative pathway, which denotes activation, and low circulating C3 levels are related to elevated values of certain hematological scores. The usual pattern of complement activation in SLE involves the classical pathway, and the alternative pathway is characterized by the activation of low-level plasma tonic C3 via hydrolysis in a process termed “tick-over”. Although difficult to interpret, we believe that complement activation via this pathway causes lower levels of lymphocytes, which would lead to higher score levels since lymphocytes are in the denominator formulas.

Unlike some other autoimmune disorders, SLE lacks established and specific biomarkers that can definitively confirm its presence or track its progression [47]. This absence of reliable biomarkers hampers early diagnosis, the accurate evaluation of disease activity, and the prediction of flare-ups. While certain biomarkers such as antinuclear antibodies and complement levels are associated with SLE, they are not exclusive to the disease and can be found in other conditions as well. This lack of specific biomarkers underscores the complexity and diversity of SLE, posing challenges for clinicians to effectively monitor and manage the disease. Consequently, there is an urgent need for further research and development of reliable biomarkers to improve the diagnosis, prognosis, and treatment of lupus [48]. We believe that our findings also dismiss, in some manner, the use of hematological scores in patients with SLE. However, their prospective role has not yet been studied.

We acknowledge the limitation that anti-cyclic citrullinated peptide autoantibodies were not assessed. This would have allowed us to rule out the presence of rheumatoid arthritis. However, patients were recruited only if they fulfilled classification criteria for SLE according to stablished guidelines. Furthermore, we also recognize that we did not collect information regarding antinuclear antibody patterns and titers. Due to this, we cannot conclude how they were associated with the blood composite scores. Additionally, we did not collect information on basophil and eosinophil numbers. However, we believe this has not affected our results. Lastly, because SLE is characterized by complexities in disease phases and flares, we cannot know how hematological scores vary longitudinally.

## 5. Conclusions

In conclusion, the hematological scores NLR, MLR, and PLR, but not SIRI, are elevated in patients with SLE compared to controls. We have not found a relationship of these scores with the activity or damage produced by the disease, which had been described in previous reports. Additionally, complement system alternative pathway activation was related to superior levels of NLR, MLR, and PLR. According to our findings, the role of this hematological composite score in assessing disease activity or damage from the disease appears to be limited.

## Figures and Tables

**Table 1:** Characteristics of systemic lupus erythematosus patients and controls.

	Controls	SLE Patients	
	(*n* = 181)	(*n* = 284)	*p*
Age, years	50 ± 12	50 ± 12	0.70
Female, *n* (%)	162 (90)	261 (92)	0.38
Body mass index, kg/m^2^	**30 ± 3**	**28 ± 6**	**<0.001**
Cardiovascular co-morbidity			
Smoking, *n* (%)	32 (17)	69 (24)	0.092
Diabetes, *n* (%)	**28** (**16**)	**16** (**6**)	**<0.001**
Hypertension, *n* (%)	**51** (**28**)	**111** (**39**)	**0.015**
Obesity, *n* (%)	49 (27)	84 (30)	0.56
Dyslipidemia, *n* (%)	140 (77)	197 (69)	0.060
Statins, *n* (%)	44 (24)	72 (25)	0.80
Aspirin, *n* (%)	**9** (**11**)	**80** (**29**)	**0.001**
Carotid intima media thickness, microns		628 ± 109	
Carotid plaque, *n* (%)		99 (36)	
SLE related data			
Disease duration, years		16 (7–24)	
CRP, mg/dL		2.0 (0.8–4.4)	
SLICC-DI		1 (0–2)	
SLICC-DI ≥ 1, *n* (%)		191 (68)	
Katz Index		2 (1–4)	
Katz ≥ 3, *n* (%)		126 (44)	
SLEDAI		2 (0–4)	
SLEDAI categories, *n* (%)			
No activity, *n* (%)		109 (40)	
Mild, *n* (%)		107 (39)	
Moderate, *n* (%)		41 (15)	
High, *n* (%)		10 (4)	
Very High, *n* (%)		4 (1)	
Auto-antibody profile			
Anti-DNA positive, *n* (%)		151 (67)	
Anti-ENA positive, *n* (%)		164 (69)	
Anti-SSA, *n* (%)		55 (35)	
Anti-SSB, *n* (%)		36 (21)	
Anti-RNP, *n* (%)		64 (28)	
Anti-Sm, *n* (%)		24 (10)	
Anti-ribosome		13 (9)	
Anti-nucleosome		32 (22)	
Anti-histone		22 (15)	
Antiphospholipid syndrome, *n* (%)		43 (16)	
Antiphospholipid autoantibodies, *n* (%)		61 (32)	
Lupus anticoagulant, *n* (%)		51 (28)	
ACA IgM, *n* (%)		22 (11)	
ACA IgG, *n* (%)		39 (20)	
Anti beta2 glycoprotein IgM, *n* (%)		19 (10)	
Anti beta2 glycoprotein IgG, *n* (%)		28 (15)	
Therapies			
Current prednisone, *n* (%)		140 (50)	
Prednisone, mg/day		5 (5–7.5)	
Hydroxychloroquine, *n* (%)		194 (69)	
Methotrexate, *n* (%)		31 (11)	
Mycophenolate mofetil, *n* (%)		31 (11)	
Azathioprine, *n* (%)		43 (15)	
Rituximab, *n* (%)		8 (3)	
Belimumab, *n* (%)		8 (3)	

Data represent mean ± SD or median (interquartile range) when data were not normally distributed. BMI: body mass index. CRP: C reactive protein. ACA: anticardiolipin. ANA: antinuclear antibodies. ENA: extractible nuclear antibodies. Anti-ENA-positive refers to being positive to any of the ENA types. SLEDAI: Systemic Lupus Erythematosus Disease Activity Index. SLEDAI categories were defined as follows: 0, no activity; 1–5, mild; 6–10, moderate; >10, high activity, >20, very high activity. SLICC: Systemic Lupus International Collaborating Clinics/American Colleague of Rheumatology Damage Index. Anti-RNP: antinuclear ribonucleoprotein antibody. Anti-SSA: anti-Sjögren’s syndrome-related antigen A. Anti-SSB: anti-Sjögren’s syndrome-related antigen B. Anti-Sm: anti-Smith antibody. Dyslipidemia was defined if one of the following was present: total cholesterol > 200 mg/dL, triglycerides > 150 mg/dL, HDL-cholesterol < 40 in men or <50 mg/dL in women, or LDL-cholesterol > 130 mg/dL. Significant *p* values are depicted in bold.

**Table 2:** Multivariable analysis of the differences between patients and controls in hematological composite scores.

	Controls	SLE Patients			
	(*n* = 181)	(*n* = 284)	*p*	Beta Coef. (95%CI)	*p*
	Univariable			Multivariable	
Neutrophils × mm^3^	**4019 ± 1496**	**2419 ± 2302**	**<0.001**	**−1755** (**−2605–**(**−905**))	**<0.001**
Lymphocytes × mm^3^	**2479 ± 874**	**1156 ± 1197**	**<0.001**	**−1057** (**−1495–**(**−619**))	**<0.001**
Monocytes × mm^3^	**582 ± 169**	**369 ± 315**	**<0.001**	**−160** (**−275–**(**−47**))	**0.006**
Eosinophils × mm^3^	**191 (110–293**)	**70** (**30–140**)	**<0.001**	**−105** (**−136–**(**−75**))	**0.015**
Basophils × mm^3^	**43 (32–61**)	**40** (**20–100**)	**<0.001**	**40** (**4–47**)	**0.020**
Platelets × 10^3^ mm^3^	**270 ± 60**	**159 ± 129**	**<0.001**	**−86** (**−132–**(**−40**))	**<0.001**
SIRI × 10^−3^	0.87 (0.63–1.32)	0.98 (0.66–1.67)	0.084	0.2 (−0.1–0.5)	0.26
NLR	**1.87 ± 1.48**	**2.33 ± 1.53**	**0.002**	**0.5** (**0.08–0.9**)	**0.020**
MLR	**0.23 (0.18–0.30**)	**0.32** (**0.24–0.42**)	**<0.001**	**0.1** (**0.02–0.2**)	**0.012**
PLR	**125 ± 73**	**161 ± 98**	**<0.001**	**45** (**10–81**)	**0.012**

In the multivariable analysis controls is considered the reference variable. Multivariable analysis is adjusted for body mass index, smoking, diabetes, hypertension, dyslipidemia, and aspirin intake. SIRI: systemic inflammation response index; NLR: neutrophil-to-lymphocyte ratio; PLR: platelet-to-lymphocyte ratio; MLR: monocyte-to-lymphocyte ratio. Significant *p* values are depicted in bold.

**Table 3:** Demographics’ and disease characteristics’ relation to blood composite scores.

							Beta Coef. (95%), *p*					
	SIRI × 10^−3^			NLR			MLR			PLR		
		*p*	p *		*p*	p *		*p*	p *		*p*	p *
Age, years	0.0009 (−0.01–0.01)	0.87		−0.003 (−0.02–0.01)	0.67		−0.001 (−0.004–0.001)	0.32		**−2** (**−3–**(**−1**))	**0.002**	
Female	**−0.8** (**−1–**(**−0.3**))	**0.001**		−1 (−1–0.1)	0.091		−0.05 (−0.2–0.05)	0.30		37 (−6–81)	0.096	
Body mass index, kg/m^2^	0.02 (−0.003–0.04)	0.082		0.02 (−0.008–0.06)	0.14		0.02 (−0.002–0.007)	0.32		1 (−1–3)	0.49	
Cardiovascular co-morbidity												
Smoking	−0.03 (−0.3–0.3)	0.85		−0.2 (−1–0.3)	0.48		−0.06 (−0.1–0.004)	0.065		**−31** (**−58–**(**−4**))	**0.023**	
Diabetes	0.1 (−1–1)	0.72		−0.2 (−1–1)	0.68		−0.08 (−0.2–0.04)	0.21		33 (−86–20)	0.22	
Hypertension	−0.06 (−0.2–0.3)	0.68		0.03 (−0.4–0.4)	0.88		0.009 (−0.05–0.07)	0.77		−17 (−41–8)	0.18	
Obesity	**0.3** (**0.04–1**)	**0.026**		0.3 (−0.07–1)	0.10		0.04 (−0.02–0.1)	0.23		22 (−1–47)	0.098	
Dyslipidemia	0.1 (−0.2–0.4)	0.41		0.09 (−0.3–0.5)	0.65		0.002 (−0.06–0.06)	0.95		−20 (−46–6)	0.12	
Statins	0.02 (−0.3–0.3)	0.90		−0.3 (−1–0.1)	0.14		−0.03 (−0.1–0.03)	0.32		**−28** (**−55–**(**−1**))	**0.040**	
Aspirin	0.3 (−0.001–1)	0.051		0.3 (−1–0.1)	0.23		0.04 (−0.02–0.1)	0.16		−4 (−30–23)	0.79	
cIMT, microns	0.0007 (−0.0005–0.002)	0.23		0.002 (−0.0001–0.003)	0.070		0.00001 (−0.0002–0.0002)	0.92		−0.05 (−0.2–0.06)	0.38	
Carotid plaque	0.2 (−0.08–0.5)	0.16		0.01 (−0.4–0.4)	0.94		0.01 (−0.04–0.07)	0.68		**−37** (**−61–**(**−12**))	**0.004**	
SLE related data												
Disease duration, years	0.005 (−0.009–0.02)	0.51		−0.005 (−0.02–0.01)	0.64		0.00001 (−0.003–0.003)	0.99		−1 (−2–0.2)	0.11	0.46
CRP, mg/dL	0.006 (−0.006–0.02)	0.35		0.003 (−0.01–0.02)	0.72		0.0002 (−0.002–0.003)	0.87		0.3 (−1–1)	0.58	
SLICC-DI	0.05 (−0.03–0.1)	0.19	0.38	0.04 (−0.08–0.2)	0.52		**0.02** (**0.002–0.03**)	**0.028**	0.11	−2 (−9–6)	0.66	
SLICC-DI ≥ 1	0.2 (−0.07–0.5)	0.14	0.48	0.1 (−0.2–0.5)	0.47		0.03 (−0.03–0.09)	0.33		−15 (−40–10)	0.25	
Katz Index	**0.09** (**0.02–0.2**)	**0.011**	**0.020**	0.08 (−0.02–0.2)	0.11	**0.039**	**0.02** (**0.007–0.04**)	**0.003**	**0.016**	1 (−5–7)	0.78	
Katz ≥ 3	**0.3** (**0.03–1**)	**0.028**	0.055	0.3 (−0.1–1)	0.15	0.057	**0.08** (**0.02–0.1**)	**0.005**	**0.020**	15 (−9–39)	0.22	
SLEDAI	−0.0004 (−0.03–0.03)	0.98		0.004 (−0.04–0.05)	0.88		0.001 (−0.006–0.008)	0.71		−0.3 (−3–3)	0.87	
SLEDAI categories												
No activity	ref.			ref.			ref.			ref.		
Mild	0.04 (−0.3–0.3)	0.82		0.2 (−0.2–0.6)	0.39		0.02 (−0.05–0.08)	0.58		−4 (−31–24)	0.79	
Moderate to very high	0.06 (−0.3–0.4)	0.77		0.2 (−0.3–0.7)	0.42		0.05 (−0.02–0.1)	0.16		10 (−23–43)	0.56	
Auto-antibody profile												
Anti-DNA positive	−0.06 (−0.4–0.3)	0.73		0.2 (−0.3–0.6)	0.47		−0.005 (−0.07–0.06)	0.87		17 (−9–43)	0.20	
ENA positive	0.1 (−0.2–0.5)	0.40		0.3 (−0.1–1)	0.19	0.27	**0.08** (**0.02–0.1**)	**0.013**	**0.024**	**40** (**11–68**)	**0.006**	**0.032**
Anti-SSA	0.2 (−0.2–1)	0.27		0.4 (−0.2–1)	0.22		0.09 (−0.002–0.2)	0.054	0.075	**43** (**5–82**)	**0.028**	**0.034**
Anti-SSB	−0.1 (−1–1)	0.89		1 (−1–2)	0.53		−0.03 (−0.3–0.3)	0.82		52 (−63–166)	0.37	
Anti-RNP	0.04 (−0.3–0.4)	0.82		0.4 (−0.1–1)	0.13	0.27	**0.1** (**0.02–0.2**)	**0.009**	**0.010**	**49** (**18–80**)	**0.002**	**0.004**
Anti-Sm	−0.08 (−0.6–0.4)	0.75		−0.09 (−1–1)	0.80		−0.008 (−0.1–0.1)	0.87		2 (−43–48)	0.93	
Anti-ribosome	0.2 (−0.5–1)	0.53		1 (−0.3–2)	0.19	0.34	0.06 (−0.1–0.2)	0.46		34 (−31–99)	0.31	
Anti-nucleosome	0.1 (−0.4–1)	0.57		1 (0.01–1)	0.047	0.099	0.1 (−0.005–0.2)	0.062	0.052	**56** (**12–101**)	**0.014**	**0.030**
Anti-histone	−0.4 (−1–0.2)	0.19	0.20	−0.5 (−1–0.3)	0.25		−0.09 (−0.2–0.04)	0.17	0.18	−17 (−69–35)	0.51	
Antiphospholipid synd.	0.28 (−0.08–0.6)	0.13	0.20	0.5 (0.02–1)	0.042	0.059	0.08 (0.009–0.2)	0.029	0.057	12 (−21–45)	0.50	
Antiphospholipid autoantibodies												
Lupus anticoagulant	0.05 (−0.3–0.4)	0.76		0.01 (−0.5–0.5)	0.96		−0.04 (−0.1–0.03)	0.28		−22 (−57–13)	0.21	
ACA IgM	−0.08 (−0.6–0.4)	0.74		−0.03 (−1–1)	0.94		−0.05 (−0.1–0.05)	0.32		−2 (−50–46)	0.94	
ACA IgG	0.16 (−0.2–0.5)	0.40		0.5 (−0.05–1)	0.073	**0.049**	0.01 (−0.06–0.09)	0.74		5 (−33–43)	0.80	
Anti beta2 glycoprotein IgM	−0.2 (−0.7–0.3)	0.49		−0.09 (−1–1)	0.82		−0.1 (−0.2–0.001)	0.053	0.059	−33 (−86–19)	0.21	
Anti beta2 glycoprotein IgG	**0.7** (**0.3–1**)	**0.001**	**0.001**	**1** (**1–2**)	**<0.001**	**<0.001**	0.08 (−0.006–0.2)	0.069	0.085	13 (−30–57)	0.54	
Current prednisone	**0.3** (**0.006–0.5**)	**0.045**	**0.037**	0.3 (−0.07–1)	0.11		**0.08** (**0.03–0.1**)	**0.004**	**0.009**	13 (−10–37)	0.27	
Prednisone, mg/day	0.06 (−0.01–0.1)	0.10	0.25	**0.1** (**0.006–0.2**)	**0.038**	**0.049**	0.01 (−0.003–0.03)	0.13	0.18	3 (−3–10)	0.33	
Hydroxychloroquine	−0.5 (−3–2)	0.68		−1 (−4–2)	0.47		0.01 (−0.4–0.5)	0.95		8 (−18–34)	0.54	
Methotrexate	−0.04 (−0.4–0.5)	0.84		0.2 (−0.4–1)	0.53		0.03 (−0.06–0.1)	0.57		23 (−16–61)	0.25	
Mycophenolate mofetil	−0.06 (−0.4–0.5)	0.77		−0.1 (−14–0.4)	0.64		0.04 (−0.05–0.1)	0.35		−18 (−56–20)	0.36	
Azathioprine	0.3 (−0.05–1)	0.089	0.065	**1** (**0.4–1**)	**<0.001**	**<0.001**	**0.2** (**0.08–0.2**)	**<0.001**	**<0.001**	**85** (**54–116**)	**<0.001**	**<0.001**
Rituximab	−0.04 (−1–1)	0.92		−0.08 (−1–1)	0.89		0.02 (−0.1–0.2)	0.83		−6 (−76–63)	0.86	
Belimumab	0.4 (−0.4–1)	0.30		1 (−0.3–2)	0.15	0.22	0.1 (−0.06–0.3)	0.20		**83** (**14–152**)	**0.018**	0.096

In this analysis, hematological scores are considered the dependent variable. BMI: body mass index; C3 C4: complement; CRP: C reactive protein; LDL: low-density lipoprotein; ACA: anticardiolipin; cIMT: carotid intima thickness; HDL: high-density lipoprotein; ANA: antinuclear antibodies; ENA: extractible nuclear antibodies; SLEDAI: Systemic Lupus Erythematosus Disease Activity Index. Anti-ENA positive refers to being positive to any of the ENA types. Anti-RNP: Antinuclear ribonucleoprotein antibody; anti-SSA: Anti-Sjögren’s syndrome-related antigen A; anti-SSB: Anti-Sjögren’s syndrome-related antigen B; Anti-Sm: anti-Smith antibody. SLEDAI categories were defined as follows: 0, no activity; 1–5, mild; 6–10, moderate; >10, high activity; >20, very high activity. SLICC: Systemic Lupus International Collaborating Clinics/American Colleague of Rheumatology Damage Index; SIRI: systemic inflammation response index; NLR: neutrophil-to-lymphocyte ratio; PLR: platelet-to-lymphocyte ratio; MLR: monocyte-to-lymphocyte ratio. * Multivariable analysis is adjusted for demographics or cardiovascular comorbidity with a *p* value univariable relation to the hematological score inferior to 0.20. SIRI adjusted for female, BMI, and aspirin. NLR adjusted for female, BMI, and statins. MLR adjusted for smoking and aspirin. PLR adjusted for age, female, smoking, hypertension, obesity, dyslipidemia, and statins. Significant *p* values are depicted in bold. To delve deeper into the relationship between disease characteristics and hematological scores, we additionally analyzed the item-one-to-one relationship of the SLICC, SLEDAI-2K, and Katz scores with each of these scores (Table 4). Regarding SLEDAI-2K items, only the presence of vasculitis (*n* = 1) and pleurisy (*n* = 4) revealed significant positive relationships with SIRI and NL, and with PLR, respectively. Within the Katz index, the low hematocrit item showed a positive association with MLR and PLR. Regarding SLICC-DI, the neuropsychiatric and peripheral vascular domains showed a negative and positive significant relationship to, respectively, PLR and MLR. The relationships of items, not domains, of SLICC-DI to hematology scores are additionally shown in Supplementary Table S1.

**Table 4:** Individual disease score items’ univariable relation to blood composite scores.

			SIRI × 10^−3^		NLR		MLR		PLR	
	*n*	%	Beta Coef. (95%). *p*		Beta Coef. (95%). *p*		Beta Coef. (95%), *p*		Beta Coef. (95%), *p*	
Katz Index										
History of cerebritis (seizure or organic brain syndrome)	12	6	0.4 (−0.02–1)	0.064	0.5 (−0.03–1)	0.064	0.06 (−0.02–0.1)	0.16	−25 (−61–11)	0.18
History of pulmonary disease	10	5	0.4 (−0.05–1)	0.086	0.3 (−0.3–1)	0.39	**0.1** (**0.06–0.2**)	**0.001**	30 (−7–66)	0.11
Biopsy proven diffuse proliferative glomerulonephritis	23	12	−0.003 (−0.3–0.3)	0.98	−0.08 (−0.5–0.3)	0.70	−0.01 (−0.07–0.05)	0.73	−13 (−38–13)	0.33
4–6 ARA criteria por SLE satisfied to date	139	73	−0.1 (−1–0.3)	0.51	−0.1 (−1–0.4)	0.63	−0.03 (−0.1–0.05)	0.44	17 (−19–52)	0.36
7 or more ARA criteria for SLE satisfied to date	23	12	0.2 (−0.07–0.5)	0.13	0.3 (−0.04–1)	0.08	**0.1** (**0.05–0.2**)	**<0.001**	16 (−9–40)	0.22
History of proteinuria (2+ or more)	62	32	0.1 (−0.3–1)	0.60	−0.05 (−1–0.5)	0.86	−0.0003 (−0.08–0.08)	0.99	1 (−34–36)	0.95
Lowest recorded hematocrit to date = 30–37%	88	46	−0.05 (−0.5–0.3)	0.81	0.05 (−0.5–1)	0.86	−0.009 (−0.09–0.07)	0.83	9 (−23–42)	0.57
Lowest recorded hematocrit to date < 30%	47	25	0.1 (−0.1–0.3)	0.26	0.1 (−0.2–0.4)	0.37	**0.06** (**0.01–0.1**)	**0.016**	**19** (**0.1–38**)	**0.049**
Highest recorded creatinine to date = 1.3–3	28	15	**0.6** (**0.1–1**)	**0.020**	0.5 (−0.2–1)	0.18	0.03 (−0.08–0.1)	0.57	−27 (−72–19)	0.25
Highest recorded creatinine to date > 3	3	2	0.2 (−1–1)	0.70	−0.1 (−1–1)	0.80	−0.01 (−0.2–0.1)	0.88	−25 (−87–37)	0.43
SLEDAI										
Seizures	1	0	−0.02 (−2–2)	0.99	−0.5 (−4–3)	0.76	−0.2 (−1–0.3)	0.48	−83 (−278–112)	0.40
Psychosis	1	0	−1 (−3–1)	0.39	−1 (−4–2)	0.41	−0.2 (−1–0.3)	0.36	−135 (−330–60)	0.17
Organic brain syndrome	0	0	-		-		-		-	
Visual disturbance	1	0	0.4 (−2–3)	0.70	2 (−1–5)	0.20	−0.09 (−1–0.4)	0.71	−122 (−317–73)	0.22
Cranial nerve disorder	1	0	−0.5 (−3–2)	0.63	−0.002 (−3–3)	0.99	−0.2 (−1–0.3)	0.41	7 (−189–203)	0.94
Lupus headache	1	0	0.04 (−2–2)	0.97	−1 (−4–2)	0.50	0.3 (−0.2–1)	0.24	57 (−138–253)	0.56
ACVA	0	0	-		-		-		-	
Vasculitis	1	0	**4** (**1–6**)	**0.001**	**4** (**1–7**)	**0.004**	0.2 (−0.3–1)	0.45	27 (−168–223)	0.78
Arthritis	9	3	−0.09 (−1–1)	0.82	0.4 (−1–1)	0.40	0.04 (−0.1–0.2)	0.59	47 (−19–113)	0.16
Myositis	0	0	-		-		-		-	
Urinary cylinders	7	3	−0.2 (−1–1)	0.70	−0.5 (−2–1)	0.42	0.1 (−0.06–0.3)	0.21	−57 (−138–23)	0.16
Hematuria	16	6	−0.3 (−1–0.3)	0.35	−0.3 (−1–1)	0.50	0.06 (−0.06–0.2)	0.31	24 (−27–77)	0.35
Proteinuria	5	2	0.3 (−1–1)	0.54	−0.1 (−2–1)	0.87	−0.02 (−0.2–0.2)	0.85	−10 (−98–79)	0.83
Pyuria	11	4	−0.06 (−1–1)	0.85	−0.3 (−1–1)	0.58	−0.08 (−0.2–0.05)	0.23	−41 (−101–19)	0.18
Rash	21	8	−0.0006 (−1–1)	0.99	−0.05 (−1–1)	0.88	0.02 (−0.09–0.1)	0.77	14 (−31–58)	0.55
Alopecia	11	4	0.3 (−0.3–1)	0.32	0.3 (−1–1)	0.55	0.07 (−0.07–0.2)	0.35	−17 (−77–44)	0.59
Mucosal ulcers	14	5	−0.06 (−1–1)	0.86	0.3 (−1–1)	0.56	0.1 (−0.03–0.2)	0.14	−1 (−61–60)	0.98
Pleurisy	3	1	−1 (−2–1)	0.41	0.2 (−2–2)	0.87	−0.07 (−0.4–0.2)	0.65	**258** (**123–393**)	**<0.001**
Pericarditis	1	0	−1 (−3–1)	0.35	−1 (−4–2)	0.40	−0.2 (−0.7–0.2)	0.31	−27 (−222–169)	0.79
Low complement	76	28	−0.05 (−0.4–0.3)	0.75	−0.09 (−1–0.3)	0.68	0.04 (−0.03–0.1)	0.26	7 (−20–34)	0.62
Elevated antiDNA	85	31	0.1 (−0.2–0.4)	0.45	0.3 (−0.1–1)	0.19	0.09 (−0.05–0.07)	0.78	20 (−6–46)	0.14
Fever	2	1	−1 (−2–1)	0.48	−0.4 (−3–2)	0.71	−0.09 (−0.4–0.2)	0.59	110 (−28–247)	0.12
Thrombopenia	10	4	0.1 (−1–1)	0.70	**1** (**0.3–2**)	**0.010**	0.03 (−0.1–0.2)	0.70	**−66** (**−129–**(**−4**))	**0.038**
Leukopenia	19	7	−0.5 (−1–0.04)	0.071	−0.004 (−1–1)	0.99	0.1 (−0.02–0.2)	0.094	**98** (**156–143**)	**<0.001**
SLICC domains										
Ocular	63	22	0.1 (−0.2–0.4)	0.56	0.06 (−0.4–1)	0.79	0.0002 (−0.07–0.07)	0.99	−8 (−37–20)	0.57
Neuropsychiatric	40	14	0.02 (−0.4–0.4)	0.91	0.07 (−0.5–1)	0.80	0.002 (−0.08–0.08)	0.97	**−47** (**−81–(−12**))	**0.008**
Renal	28	10	0.1 (−0.3–1)	0.67	−0.2 (−1–0.4)	0.55	−0.005 (−0.1–0.09)	0.92	−25 (−64–14)	0.21
Pulmonary	19	7	−0.1 (−1–0.4)	0.68	−0.3 (−1–0.5)	0.43	0.05 (−0.06–0.2)	0.40	7 (−41–56)	0.77
Cardiovascular	23	8	−0.03 (−1–0.5)	0.90	−0.07 (−1–1)	0.86	0.06 (−0.05–0.2)	0.32	0.3 (−47–48)	0.99
Peripheral vascular	34	12	0.2 (−0.2–1)	0.38	0.3 (−0.3–1)	0.30	**0.1** (**0.02–0.2**)	**0.020**	13 (−24–50)	0.50
Gastrointestinal	28	10	−0.1 (−1–0.3)	0.56	−0.3 (−1–0.3)	0.34	0.03 (−0.06–0.1)	0.51	−19 (−59–21)	0.35
Musculoskeletal	89	31	0.002 (−0.3–0.3)	0.99	0.2 (−0.2–1)	0.41	0.03 (−0.03–0.09)	0.36	18 (−7–43)	0.17
Skin	39	14	0.03 (−0.4–0.4)	0.88	−0.3 (−1–0.2)	0.24	0.05 (−0.03–0.1)	0.22	−16 (−50–19)	0.38
Premature gonadal failure	19	7	−0.02 (−1–1)	0.94	0.05 (−1–1)	0.90	−0.02 (−0.1–0.1)	0.79	5 (−45–56)	0.83
Diabetes (regardless of treatment)	18	6	0.003 (−1–1)	0.99	−0.3 (−1–0.5)	0.42	−0.09 (−0.2–0.03)	0.14	−40 (−90–10)	0.11
Malignancy (excluded dysplasia)	11	4	−1 (−1–0.2)	0.14	−1 (−2–0.2)	0.13	−0.1 (−0.3–0.01)	0.066	−18 (−84–48)	0.60

History of pulmonary disease refers to the presence of lupus pneumonitis, pulmonary hemorrhage, or pulmonary hypertension. SLEDAI: Systemic Lupus Erythematosus Disease Activity Index. SLE: Systemic Lupus Erythematosus. SLICC: Systemic Lupus International Collaborating Clinics/American Colleague of Rheumatology Damage Index. The presence of a SLICC domain involvement if shown if the point in the domain is ≥1. See Supplementary Table S1. ARA: American Rheumatism Association. ACVA: acute cerebrovascular accident. SIRI: systemic inflammation response index. NLR: neutrophil-to-lymphocyte ratio; PLR: platelet-to-lymphocyte ratio; MLR: monocyte-to-lymphocyte ratio. Significant *p* values are depicted in bold.

**Table 5:** Univariable analysis of the relation of complement system pathways and components to blood scores in SLE patients.

		Beta Coef. (95%), *p*							
		SIRI × 10^−3^		NLR		MLR		PLR	
Classical pathway									
Functional assay, %	91 ± 38	0.008 (−0.003–0.004)	0.64	−0.004 (−0.009–0.001)	0.11	−0.0002 (−0.001–0.0005)	0.54	**−0.5** (**−0.9–**(**−0.2**)	**0.001**
C1q, mg/dL	34 ± 11	−0.004 (−0.02–0.009)	0.52	−0.0003 (−0.02–0.02)	0.97	−0.001 (−0.004–0.001)	0.30	−0.4 (−2–0.07)	0.46
Lectin pathway									
Functional assay, %	10 (1–41)	0.002 (−0.001–0.005)	0.28	0.002 (−0.003–0.006)	0.43	0.0002 (−0.0005–0.0009)	0.53	−0.2 (−0.5–0.07)	0.14
Common elements of the classic and lectin pathways									
C2, mg/dL	2.5 ± 1.2	−0.03 (−0.1–0.09)	0.61	−0.1 (−0.3–0.04)	0.14	−0.02 (−0.05–0.001)	0.065	−3 (−13–7)	0.58
C4, mg/dL	21 ± 12	−0.0007 (−0.01–0.01)	0.90	−0.01 (−0.03–0.005)	0.19	−0.002 (−0.005–0.00002)	0.052	**−1** (**−2–**(**−0.2**)	**0.024**
C1 inhibitor, mg/dL	32 ± 9	0.01 (−0.003–0.03)	0.11	0.02 (−0.008–0.04)	0.19	0.0006 (−0.003–0.004)	0.72	−0.4 (−2–1)	0.63
Alternative pathway									
Functional assay, %	41 (12–79)	−0.002 (−0.006–0.001)	0.19	**−0.007** (**−0.01 −** (**−0.002**)	**0.007**	**−0.001** (**−0.002 −** (**−0.0007**)	**<0.001**	**−0.8** (**−1–**(**−0.4**)	**<0.001**
Factor D, ng/ml	2593 ± 1836								
Common elements of the three pathways									
C3, mg/dL	130 ± 40	−0.0007 (−0.004–0.003)	0.69	−0.004 (−0.009–0.001)	0.14	**−0.0007** (**−0.001** (**−0.00002**)	**0.043**	**−0.4** (**−0.7–**(**−0.06**)	**0.020**
C3a, mg/dL	39 ± 10	0.003 (−0.01–0.02)	0.67	−0.003 (−0.02–0.02)	0.76	−0.002 (−0.005–0.0006)	0.13	−0.2 (−1–1)	0.75
Factor H, ng/mL × 10^−3^	389 (281–564)	−0.00008 (−0.0003–0.0001)	0.40	−0.00007 (−0.0003–0.0002)	0.59	−0.00002 (−0.00005–0.00001)	0.26	0.001 (−0.01–0.02)	0.85

Data represent means ± SD or median (interquartile range) when data were not normally distributed. Complement routes and elements are considered the independent variable. SIRI: systemic inflammation response index; NLR: neutrophil-to-lymphocyte ratio; PLR: platelet-to-lymphocyte ratio; MLR: monocyte-to-lymphocyte ratio. Significant *p* values are depicted in bold.

## Data Availability

The data sets used and/or analyzed in the present study are available from the corresponding author upon request.

## Supplementary Materials

The following supporting information can be downloaded at: https://www.mdpi.com/article/10.3390/biomedicines11102782/s1, Supplementary Table S1: Relationship of SLICC score items to blood composite scores.
