# Supplementary material for: Blood Composite Scores in Patients with Systemic Lupus Erythematosus
Source: Biomedicines. 2023 Oct 13;11(10):2782. doi: 10.3390/biomedicines11102782 (PMC10604879; doi:10.3390/biomedicines11102782)
Supplement: Supplementary file 1 [file biomedicines-11-02782-s001.zip › biomedicines-2557295-supplementary.pdf]

biomedicines

MDPI

Supplementary Table S1. Relationship of SLICC score items to blood composite scores

	n	%	SIRI beta coef. (95%).	p	NLR beta coef. (95%).	p	MLR beta coef. (95%).	p	PLR beta coef. (95%).	p
Ocular										
Any cataract ever	29	11	0.4 (-0.02-0.8)	0.061	0.4 (-0.2-1)	0.18	0.06 (-0.03-0.2)	0.18	4 (-34-42)	0.83
Retinal change or optic atrophy	33	12	0.2 (-0.2-0.6)	0.34	0.09 (-1-1)	0.78	0.02 (-0.07-0.1)	0.65	-23 (-60-15)	0.24
Points =>1 in the domain	63	22	0.1 (-0.2-0.4)	0.56	0.06 (-0.4-1)	0.79	0.0002 (-0.07-0.07)	0.99	-8 (-37-20)	0.57
Neuropsychiatric										
Cognitive impairment	7	3	-0.05 (-0.9-0.8)	0.90	-0.4 (-2-1)	0.53	-0.03 (-0.2-0.1)	0.71	-58 (-133-16)	0.12
Seizures requiring therapy for 6 months	15	5	0.5 (-0.1-1)	0.11	1 (-0.02-2)	0.057	0.1 (-0.02-0.2)	0.11	-42 (-97-13)	0.13
Cerebrovascular accident ever	13	5	-0.2 (-1-0.4)	0.60	-0.03 (-1-1)	0.95	-0.07 (-0.2-0.05)	0.95	-24 (-75-28)	0.95
Cranial or peripheral neuropathy	5	2	-0.4 (-2-1)	0.55	-0.4 (-2-1)	0.66	-0.04 (-0.3-0.2)	0.77	-31 (-144-82)	0.59
Transverse myelitis	1	0	-0.1 (-2-2)	0.90	-1 (-4-2)	0.64	-0.1 (-1-0.4)	0.69	-91 (-286-103)	0.36
Points =>1 in the domain	40	14	0.02 (-0.4-0.4)	0.91	0.07 (-0.5-1)	0.80	0.002 (-0.08-0.08)	0.97	**-47 (-81-(-12))**	**0.008**
Renal										
Estimated or glomerular filtration rate <50%	13	5	0.08 (-0.6-1)	0.80	-0.3 (-1-1)	0.46	0.02 (-0.1-0.1)	0.82	-24 (-82-33)	0.41
Proteinuria 3.5 gm/24 hours	7	3	0.3 (-0.5-1)	0.49	-0.1 (-1-1)	0.82	-0.04 (-0.2-0.1)	0.63	-41 (-116-34)	0.29
End-stage renal disease	4	1	0.1 (-0.2-0.5)	0.50	-0.2 (-1-0.3)	0.42	0.004 (-0.07-0.08)	0.92	-18 (-51-15)	0.28
Points =>1 in the domain	28	10	0.1 (-0.3-1)	0.67	-0.2 (-1-0.4)	0.55	-0.005 (-0.1-0.09)	0.92	-25 (-64-14)	0.21
Pulmonary										
Pulmonary hypertension	4	1	-0.2 (-1-1)	0.80	1 (-1-2)	0.56	0.2 (-0.08-0.4)	0.17	87 (-22-195)	0.12
Pulmonary fibrosis	4	1	1 (-0.5-2)	0.26	-0.4 (-2-1)	0.64	0.2 (-0.07-0.4)	0.19	-11 (-106-84)	0.82
Shrinking lung	2	1	-0.4 (-2-1)	0.62	-1 (-3-2)	0.60	-0.03 (-0.3-0.3)	0.87	-35 (-168-98)	0.61
Pleural fibrosis	1	0	0.1 (-2-2)	0.94	-0.2 (-3-3)	0.92	-0.06 (-1-0.4)	0.78	-82 (-270-107)	0.29
Pulmonary infarction	1	0	-1 (-3-1)	0.45	-1 (-4-2)	0.43	-0.2 (-1-0.3)	0.48	-41 (-227-145)	0.66
Points =>1 in the domain	19	7	-0.1 (-1-0.4)	0.68	-0.3 (-1-0.5)	0.43	0.05 (-0.06-0.2)	0.40	7 (-41-56)	0.77
Cardiovascular										
Angina or coronary artery bypass	4	1	0.2 (-1-1)	0.74	0.4 (-1-2)	0.59	0.04 (-0.2-0.3)	0.70	-31 (-129-68)	0.54
Myocardial infarction ever	2	1	0.3 (-1-2)	0.67	-0.03 (-2-2)	0.98	0.05 (-0.4-0.3)	0.77	-78 (-216-60)	0.27
Cardiomyopathy	2	1	-0.4 (-3-2)	0.69	-0.3 (-3-3)	0.84	0.3 (-0.2-1)	0.23	**260 (67-453)**	**0.008**
Valvular disease	9	3	0.001 (-1-1)	0.99	-0.01 (-1-1)	0.98	0.04 (-0.1-0.2)	0.68	-4 (-85-77)	0.92
Pericarditis for 6 months, or pericardiectomy	2	1	1 (-0.5-3)	0.17	0.3 (-2-2)	0.82	0.2 (-0.1-1)	0.18	18 (-120-156)	0.80

Points =>1 in the domain	23	8	-0.03 (-1-0.5)	0.90	-0.07 (-1-1)	0.86	0.06 (-0.05-0.2)	0.32	0.3 (-47-48)	0.99
Peripheral vascular										
Claudication for 6 months	3	1	0.1 (-1-1)	0.82	0.3 (-1-2)	0.73	-0.1 (-0.4-0.2)	0.49	-31 (-145-83)	0.59
Minor tissue loss (pulp space)	5	2	0.1 (-1-1)	0.82	-0.3 (-2-1)	0.68	**0.3 (0.1-1)**	**0.003**	-11 (-110-88)	0.82
Significant tissue loss ever	0	0	-	-	-	-	-	-	-	-
Venous thrombosis	14	5	-0.06 (-1-1)	0.85	0.1 (-1-1)	0.76	**0.2 (0.04-0.3)**	**0.010**	24 (-34-82)	0.42
Points =>1 in the domain	34	12	0.2 (-0.2-1)	0.38	0.3 (-0.3-1)	0.30	**0.1 (0.02-0.2)**	**0.020**	13 (-24-50)	0.50
Gastrointestinal										
Infarction or resection of bowel	22	8	-0.1 (-0.6-0.4)	0.69	-0.3 (-1-0.4)	0.36	0.04 (-0.06-0.1)	0.45	-18 (-63-27)	0.42
Mesenteric insufficiency	1	0	-0.3 (-2-2)	0.79	1 (-2-4)	0.54	**1 (1-2)**	**<0.001**	-	-
Infarction or resection of bowel below duodenum, spleen, liver, or chronic peritonitis	1	0	-0.4 (-3-2)	0.70	-0.5 (-3-3)	0.75	-0.1 (-1-0.4)	0.63	-50 (-245-144)	0.61
Stricture or upper gastrointestinal tract surgery ever	0	0	-	-	-	-	-	-	-	-
Pancreatic insufficiency	0	0	-	-	-	-	-	-	-	-
Points =>1 in the domain	28	10	-0.1 (-1-0.3)	0.56	-0.3 (-1-0.3)	0.34	0.03 (-0.06-0.1)	0.51	-19 (-59-21)	0.35
Musculoskeletal										
Muscle atrophy or weakness	3	1	**2 (1-3)**	**0.002**	**2 (0.06-4)**	**0.043**	0.1 (-0.2-0.4)	0.50	-29 (-139-81)	0.6
Deforming or erosive arthritis	40	15	-0.3 (-1-0.1)	0.18	0.03 (-0.5-1)	0.92	0.06 (-0.02-0.1)	0.18	**44 (12-77)**	**0.007**
Osteoporosis with fracture	23	9	0.02 (-0.5-1)	0.92	0.03 (-1-1)	0.92	-0.02 (-0.1-0.09)	0.76	-16 (-58-26)	0.46
Avascular necrosis	7	3	0.04 (-1-1)	0.93	-0.3 (-2-1)	0.58	0.007 (-0.2-0.2)	0.94	-37 (-115-41)	0.36
Osteomyelitis	1	0	0.4 (-2-3)	0.70	2 (-1-5)	0.18	-0.08 (-1-0.4)	0.73	-119 (-308-70)	0.22
Tendon rupture	4	2	-0.1 (-1-1)	0.86	-0.2 (-2-1)	0.84	0.06 (-0.2-0.3)	0.58	51 (-44-146)	0.29
Points =>1 in the domain	89	31	0.002 (-0.3-0.3)	0.99	0.2 (-0.2-1)	0.41	0.03 (-0.03-0.09)	0.36	18 (-7-43)	0.17
Skin										
Scarring chronic alopecia	16	6	0.1 (-1-1)	0.78	-0.2 (-1-1)	0.72	-0.02 (-0.1-0.1)	0.77	-32 (-86-22)	0.24
Extensive scarring or panniculitis	10	4	1 (-0.1-1)	0.094	0.3 (-1-1)	0.60	**0.2 (0.05-0.4)**	**0.010**	41 (-25-108)	0.22
Skin ulceration	4	1	0.4 (-1-2)	0.47	0.1 (-1-2)	0.86	0.2 (-0.06-0.4)	0.15	-5 (-104-94)	0.93
Points =>1 in the domain	39	14	0.03 (-0.4-0.4)	0.88	-0.3 (-1-0.2)	0.24	0.05 (-0.03-0.1)	0.22	-16 (-50-19)	0.38
Premature gonadal failure	19	7	-0.02 (-1-1)	0.94	0.05 (-1-1)	0.90	-0.02 (-0.1-0.1)	0.79	5 (-45-56)	0.83
Diabetes (regardless of treatment)	18	6	0.003 (-1-1)	0.99	-0.3 (-1-0.5)	0.42	-0.09 (-0.2-0.03)	0.14	-40 (-90-10)	0.11
Malignancy (exclude dysplasia)	11	4	-1 (-1-0.2)	0.14	-1 (-2-0.2)	0.13	-0.1 (-0.3-0.01)	0.066	-18 (-84-48)	0.60

SLICC items and domains represent the independent variable. Significant p values are depicted in bold.
SLICC: Systemic Lupus International Collaborating Clinics/American Colleague of Rheumatology Damage Index.
SIRI: systemic inflammation response index; NLR: neutrophil-to-lymphocyte ratio; PLR: platelet-to-lymphocyte ratio; MLR: monocyte-to-lymphocyte ratio.
